# Sensitivity of National Healthcare Safety Network definitions to capture healthcare-associated transmission identified by whole-genome sequencing surveillance

**DOI:** 10.1017/ice.2023.52

**Published:** 2023-03-28

**Authors:** Alexander J. Sundermann, Joseph Penzelik, Ashley Ayres, Graham M. Snyder, Lee H. Harrison

**Affiliations:** 1 Microbial Genomic Epidemiology Laboratory, Center for Genomic Epidemiology, University of Pittsburgh, Pittsburgh, Pennsylvania; 2 Division of Infectious Diseases, University of Pittsburgh School of Medicine, Pittsburgh, Pennsylvania; 3 Department of Infection Control and Hospital Epidemiology, UPMC Presbyterian, Pittsburgh, Pennsylvania; 4 Department of Epidemiology, School of Public Health, University of Pittsburgh, Pittsburgh, Pennsylvania

## Abstract

The National Healthcare Safety Network (NHSN) definitions are critical for standardizing healthcare-associated infection surveillance in US healthcare facilities. However, their use in accurately detecting healthcare-associated transmission (HAT) has not been measured. Using whole-genome sequencing surveillance data, we show that the NHSN has a sensitivity of 44.4% in detecting HAT.

The Centers for Disease Control and Prevention (CDC) National Healthcare Safety Network (NHSN) is a vital tool for healthcare infection prevention personnel to track and report healthcare-associated infections (HAIs) across the United States. Using standardized surveillance definitions of infections, it enables infection preventionists to guide patient harm reduction in their facility. The CDC utilizes these infection data to provide summaries of HAI trends that influence and direct future interventions. Although the NHSN patient-safety component was intended for HAI surveillance and quality improvement, it is one of the many tools that infection preventionists use to identify outbreaks or to conduct case finding within an outbreak.^1–4^ However, the accuracy of NHSN definitions to identify transmission of exogenous pathogens is not fully understood.

WGS surveillance is an emerging approach that can uncover undetected healthcare transmission and often excludes suspected outbreaks that are detected using traditional infection prevention methods. Our institution has recently studied the impact of WGS surveillance using the Enhanced Detection System for Healthcare-Associated Transmission (EDS-HAT), and we have previously described its potential to detect otherwise unidentified outbreaks.^5–8^ Here, we describe the sensitivity of the NHSN HAI definitions to detect healthcare-associated transmission and outbreaks of bacterial pathogens using EDS-HAT as the gold standard.

## Methods

### Study setting

This study was performed based on data from November 2016 to November 2018 at the University of Pittsburgh Medical Center–Presbyterian Hospital (UPMC), an adult tertiary-care hospital with 758 total beds and 134 critical care beds. Each year, >400 solid-organ transplants are performed here. Ethics approval for this study was obtained from the University of Pittsburgh Institutional Review Board.

### Transmission identified by EDS-HAT

The inclusion criteria for selecting isolates for WGS surveillance have been described previously.^8^ Briefly, clinical isolates for select bacterial pathogens were sequenced if the patient had been an inpatient for ≥3 days or had had a healthcare exposure in the prior 30 days. Isolates were considered part of a hospital outbreak using 15 single-nucleotide polymorphism (SNP) thresholds for all organisms except *Clostridioides difficile* (2 SNPs). For this analysis, we conservatively deemed the first case detected in an outbreak or transmission event as not a healthcare transmission given that the first case could potentially be community-associated case that was then transmitted in the healthcare setting to subsequent patients.

### NHSN characterization and sensitivity calculations

Infection preventionists utilized the 2016–2018 *NHSN Patient Safety Component Manuals* to determine whether the infections were deemed present on admission (signs or symptoms before hospitalization day 3) or were hospital onset and met the surveillance definition. These data were recorded by infection preventionists in a surveillance software system (TheraDoc version 5.2.0.HF1.42, Salt Lake City, UT). Investigators searched the NHSN HAI data set for matching infection events by first searching by patient and then by bacterial species with an NHSN HAI date 14 days before or after the EDS-HAT isolate date. The sensitivity of NHSN case definitions to detect transmission-associated infections was calculated as a percentage using the number of patients identified as part of a hospital outbreak or transmission by EDS-HAT as the denominator and the number of those patients who were considered to have an NHSN-defined HAI as the numerator.

## Results

### Healthcare-associated transmission

From November 2016 to November 2018, 2,752 unique patient isolates were sequenced.^8^ Among them, 297 isolates, including 198 (66.7%) second or subsequent isolates, comprised 99 clusters of genetically related pathogens defining outbreaks. Therefore, 297 was the denominator for determining the sensitivity of NHSN case definitions.

### NHSN characterization

**Fig. 1.**
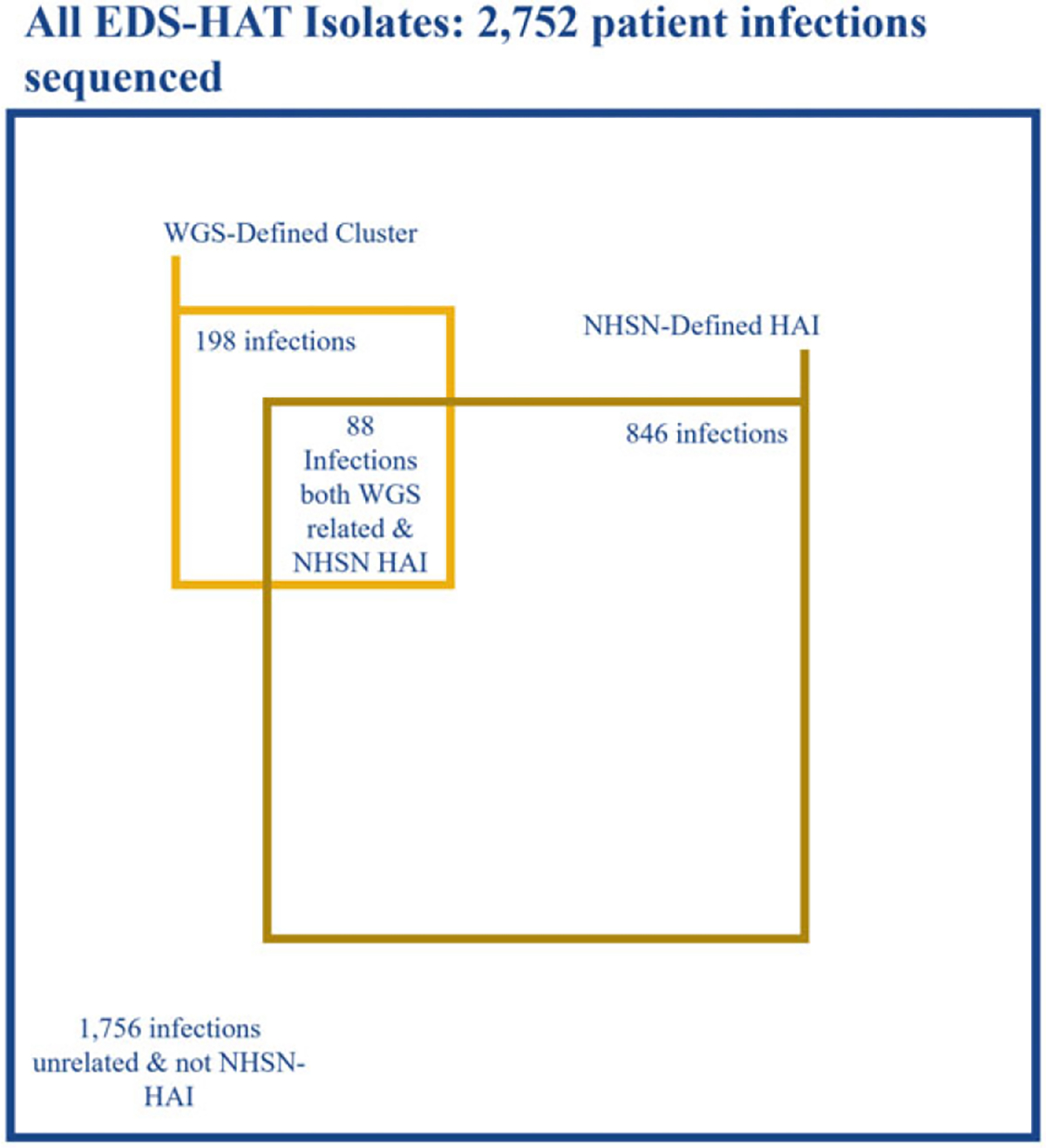
Distribution of WGS and NHSN characterization. Note. EDS-HAT, enhanced-detection system for healthcare-associated transmission; WGS, whole-genome sequencing; NHSN, National Healthcare Safety Network; HAI, healthcare-associated Infection. Figure to scale.

Of the 99 excluded first isolates in a cluster, 45 (45.5%) met the definition of a hospital-onset NHSN infection. Of the remaining 198 outbreak isolates, 88 were deemed hospital onset by the NHSN, which yielded a sensitivity of 44.4% to detect healthcare-associated transmission (Fig. 1). The predominant NHSN infection type determined to be part of an outbreak by WGS was *C. difficile* infection, followed by surgical-site infections (Supplementary Table S1 online). Excluding *C. difficile* would have yielded a sensitivity of 33.1% for the NHSN to detect healthcare-associated transmission. Of those isolates deemed to be part of an outbreak but that did not meet NHSN case definitions, the most common specimen sources were respiratory samples and urine (29 isolates each) followed by wounds (Table 1). The predominant reason for an infection not meeting the NHSN hospital-onset definition was most commonly being present on admission (58.2%), followed by not meeting the infection definition criteria (42%).

## Discussion

In this study, the sensitivity of NHSN hospital-onset infection definitions to accurately detect healthcare-associated transmission as defined by WGS surveillance was only 44.4%. Although the NHSN is a valuable tool for tracking HAIs, healthcare institutions should consider adopting WGS surveillance in addition to other outbreak detection methodologies.

Most patients with WGS-defined outbreak strains did not meet NHSN hospital-onset criteria because their infection was deemed to be present on admission. An infection is considered to have been present on admission if the signs or symptoms occur within the first 2 hospital days or prior to admission. Our collection criteria for EDS-HAT includes a prior healthcare exposure within the prior 30 days from admission regardless of the presence of signs or symptoms on the first 2 days of admission. This finding possibly indicates that these patients acquired their infection in a prior healthcare exposure, were discharged, and re-presented to our hospital with the infection.

A reliable surveillance program for outbreak detection should be sensitive for detecting hospital transmission. This study and past findings indicate that WGS surveillance can substantially enhance the detection of hospital transmission.^5–8^ Real-time WGS surveillance can often refute the presence of an outbreak and detect previously undetected outbreaks.^9^ More broadly applied, WGS surveillance has the potential to change outbreak and transmission detection for infection prevention.

Our study had several limitations. First, the criteria used to select isolates for EDS-HAT sequencing may omit infections that are truly hospital acquired. However, these instances are likely rare because isolates were included if they were collected in the first 2 days of admission but a patient had had a healthcare exposure in the 30 days prior to admission. Second, our NHSN infection surveillance was performed by manual review. However, all of our NHSN data underwent review by infection preventionists and study investigators to ensure accuracy. Third, this study was performed at a single-center, tertiary-care hospital; therefore, our results may not be generalizable to all US hospitals. Fourth, our inclusion criteria for WGS did not include all patient infections and thus may not be a true measurement of specificity. However, our criteria for sequencing targets potential HAIs, which is most representative of the population of concern. Fifth, there is no gold standard for determining transmission. WGS is the closest available tool for determining transmission. Sixth, many WGS-defined transmissions were from patients who were discharged from the hospital and re-presented with the infection. We cannot exclude common community sources as the result of this infection. Lastly, we only compared WGS surveillance to NHSN definitions in detecting outbreaks. Future studies should analyze WGS surveillance against other detection methods.

In conclusion, we have shown that the NHSN patient-safety component has low sensitivity to detect healthcare-associated transmission, as identified by WGS surveillance. Incorporation of WGS surveillance into HAI surveillance systems would enable healthcare institutions to better capture healthcare-associated transmission, to implement efforts to reduce them, and to improve patient safety.

## Supplementary Material

Sundermann et al. supplementary material

## Figures and Tables

**Table 1.** Infection Sources Determined to be Part of an Outbreak by WGS Surveillance and Not Hospital-Onset by NHSN

Isolate Source	Total	Present on Admission	Did Not Meet Criteria
Respiratory	29	5	24
Urine	29	14	15
Wound	22	15	7
Stool	17	17	0
Blood	13	13	0
Total	110	64 (58.2%)	46 (42%)

Note. WGS, whole genome sequencing; NHSN, National Healthcare Safety Network.
