# Supplementary material for: Sensitivity of National Healthcare Safety Network definitions to capture healthcare-associated transmission identified by whole-genome sequencing surveillance
Source: Infect Control Hosp Epidemiol. 2023 Mar 28;44(10):1663–5. doi: 10.1017/ice.2023.52 (PMC10533730; doi:10.1017/ice.2023.52)
Supplement: Supplementary file 1 [file S0899823X23000521sup001.docx]

**TABLES**

**Table S1**. Infections that were determined to be both part of an outbreak by WGS surveillance and hospital-onset by NHSN

| **Category** | **Infection Type** | **No. (%) Isolates** |
| --- | --- | --- |
| Major Infection Types | CLABSI | 4 (4.5) |
|  | GI-CDI | 42 (47.7) |
|  | SSI | 12 (13.6) |
|  | UTI-CAUTI | 1 (1.1) |
|  | VAE | 5 (5.7) |
|  | PNEU | 4 (4.5) |
| Other Infection Types | CVS-MED | 1 (1.1) |
|  | EENT-CONJ | 1 (1.1) |
|  | GI-IAB | 2 (2.3) |
|  | LCBI - Non-CLABSI | 4 (4.5) |
|  | LRI-LUNG | 2 (2.3) |
|  | SST-ST | 5 (5.7) |
|  | UTI-SUTI | 5 (5.7) |
| **Total** |  | **88** |

WGS: Whole Genome Sequencing; NHSN: National Healthcare Safety Network; CLABSI, Central Line-associated Bloodstream Infection; GI, Gastrointestinal System Infection; CDI, Clostridioides difficile infection; UTI, Urinary Tract Infection; CAUTI, Catheter-associated Urinary Tract Infection; VAE, Ventilator-associated event; PNEU, Non-ventilator Associated Pneumonia; CVS, Cardiovascular System Infection; MED: Mediastinitis; EENT, Eye, Ear, Nose, Throat, or Mouth Infection; CONJ: Conjunctivitis; LCBI, Laboratory Confirmed Bloodstream Infection (non-central line associated & non-mucosal barrier injury); LRI-LUNG, Lower Respiratory Infection, Other Than Pneumonia; SST-ST, Skin and Soft Tissue Infection, Soft Tissue
